# Trends in research related to fetal therapy from 2012 to 2022: a bibliometric analysis

**DOI:** 10.3389/fped.2023.1288660

**Published:** 2024-01-04

**Authors:** Yang Jia, Xiaoling Liang, Lini Liu, Huixi Ma, Chenhao Xu, Jingyuan Zeng, Rong Xu, Lu Ye, Linjun Xie

**Affiliations:** ^1^ Department of Radiology, Key Laboratory of Obstetric & Gynecologic and Pediatric Diseases and Birth Defects of Ministry of Education, West China Second University Hospital, Sichuan University, Chengdu, Sichuan, China; ^2^ Department of Ultrasound, Sichuan Provincial Maternity and Child Health Care Hospital, Chengdu, Sichuan, China; ^3^ West China School of Medicine, Sichuan University, Chengdu, Sichuan, China; ^4^ Department of Ultrasound, Key Laboratory of Obstetric & Gynecologic and Pediatric Diseases and Birth Defects of Ministry of Education, West China Second University Hospital, Sichuan University, Chengdu, Sichuan, China

**Keywords:** fetal therapy, bibliometric analysis, trend, CiteSpace, VOSviewer

## Abstract

**Background:**

The development of prenatal diagnosis technology allows prompt detection of severe fetal diseases. To address adverse factors that threaten fetal survival, fetal therapy came into existence, which aims to preserve the function after birth to a higher degree and improve the quality of life.

**Objective:**

To conduct a comprehensive bibliometric analysis of studies on fetal therapy in the past decade and explore the research trends and hotspots in this field.

**Methods:**

We conducted a systematic search on the Web of Science Core Collection to retrieve studies related to fetal therapy published from 2012 to 2022. VOSviewer and CiteSpace were used to analyze the key features of studies, including annual output, countries/regions, institutions, authors, references, research hotspots, and frontiers.

**Results:**

A total of 9,715 articles were included after eliminating duplicates. The annual distribution of the number of articles showed that the number of articles published in fetal therapy had increased in the past decade. Countries and institutions showed that fetal therapy is more mature in the United States. Author analysis showed the core investigators in the field. Keyword analysis showed the clustering and emergence frequency, which helped summarize the research results and frontier hotspots in this field. The cocited references were sorted out to determine the literature with a high ranking of fetal therapy in recent years, and the research trend in recent years was analyzed.

**Conclusions:**

This study reveals that countries, institutions, and researchers should promote wider cooperation and establish multicenter research cooperation in fetal therapy research. Moreover, fetal therapy has been gradually explored from traditional surgical treatment to gene therapy and stem cell therapy. In recent years, fetoscopic laser surgery, guideline, and magnetic resonance imaging have become the research hotspots in the field.

## 1 Introduction

With the rapid development of prenatal ultrasound diagnosis, molecular genetics, and interventional prenatal diagnosis techniques, many fetal diseases can be screened and diagnosed during the prenatal period (1). Neonatal and pediatric subspecialties have rapidly emerged, and most fetal diseases are well diagnosed before delivery, particularly isolated fetal structural abnormalities that can be corrected after birth (2). However, for a small number of severe fetal diseases, due to their rapid progression and poor prognosis, effective postpartum treatment is still lacking, which has greatly promoted the development of fetal therapy (3). Fetal therapy involves interventions administered during pregnancy to stop the progression of fetal diseases (4). Fetal therapy intends to prevent further disease progression and create conditions for postnatal treatment to reduce perinatal infant mortality and improve their quality of life (5). Fetal therapy is performed during pregnancy and involves invasive procedures; thus, some risks are unavoidable. In 1982, the International Society of Fetal Medicine and Surgery (IFMSS) first proposed principles that must be followed in fetal treatment to regulate the indications and procedures for treatment (6). Subsequently, in 2017, the North American Fetal Therapy Network and IFMSS members revised and supplemented the 1982 Fetal Therapy Basic Principles to promote further development in the field (7). They constructively suggested that fetal therapy was no longer limited to improving survival rates but increasingly focused on maternal reproductive health. Researchers should focus on reducing morbidity, improving the long-term prognosis of patients, balancing maternal and fetal risks associated with treatment, and establishing multidisciplinary teams. Fetal treatment is the reversal of pathological changes, restoration of normal anatomy, or preservation of physiological functions under prenatal intervention. Therefore, understanding the current status, main research institutions, and hotspots of fetal therapy will help with further research on the occurrence and development, treatment, and long-term prognosis of fetal diseases.

The gradual emergence of bibliometric analysis in recent years has brought a certain simplicity to summarizing the main content and cutting-edge hotspots in the research field. Bibliometric analysis is a quantitative analysis that combines mathematical and statistical methods, and it can help researchers understand the characteristics of field development over time (8). An in-depth assessment of research trends and focus on a particular topic are possible, and the results of the bibliometric evaluation can provide recommendations for further research and decision making. CiteSpace and VOSviewer were used to analyze fetal therapy, and their combination can obtain an intuitive visual network diagram in this field. To reveal the structure of the research domain, CiteSpace visual analysis generates cocitation networks based on reference citations (9).

Most of the existing reviews of fetal therapy summarize the status quo of treatment methods and fetal diseases; however, a macrosummary and induction of the whole field of fetal therapy remain unknown. In recent years, CiteSpace and VOSviewer have been widely used in many fields. Currently, targeted bibliometric analysis of scientific research on fetal therapy worldwide is lacking (10). Therefore, this study aimed to review the basics, hotspots, and frontiers of fetal therapy through bibliometric analysis.

## 2 Materials and methods

### 2.1 Data source and search strategy

**Figure 1:**
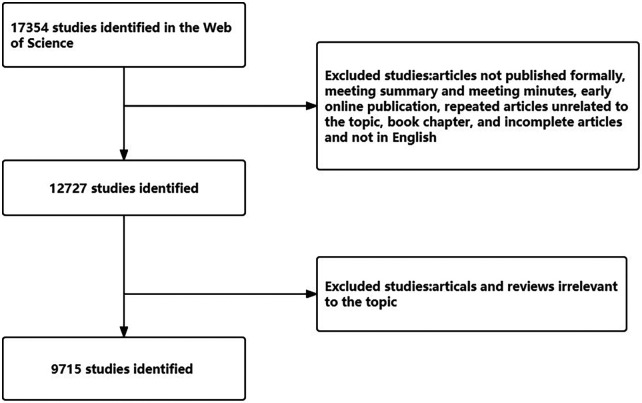
Flow diagram of the screening process used to identify publications about fetal therapy.

All published articles were available through January 14, 2023 at the Science Core Collection to eliminate large errors caused by routine database changes. The core collection of the Web of Science (WOS) was used as the data source. The search strategy used was as follows: [TS = (“fetal therapies” OR “fetal therapy” OR “fetal surgery” OR “fetoscopy” OR “blood transfusion, intrauterine” OR “intrauterine transfusion” OR “ex utero intrapartum technique procedures”)] AND [Language = (English)] AND [document type = (“article” OR “review”)], and the retrieval time was limited from January 1, 2012 to December 31, 2022. The following exclusion criteria were used to screen the obtained study: articles not published formally, meeting summaries and meeting minutes, early online publications, repeated articles unrelated to the topic, book chapters, and incomplete articles. Figure 1 provides detailed information on enrollment and selection. Because data were retrieved directly from the database, ethical approval was not needed.

### 2.2 Data analysis tools and statistical methods

The included studies were analyzed using VOSviewer and CiteSpace (6.1.R6) software. VOSviewer, a bibliometric software with strong graphical capabilities, is suitable for processing large-scale data and can be used to construct relational networks and data visualization (11). Using a visual map created by VOSviewer, nodes represent countries, institutions, authors, or keywords, which can be connected via coauthorship, citation, co-occurrence, and cocitation analysis. The size of the node was determined by the weight of the element, such as the number of publications, citations, or frequency of occurrence. Each node was assigned a color, and the same color represented the same cluster, which was a set of items in the network with similar properties. The link between nodes represented the correlation between elements, and the thickness of the link represented the strength of the link. Total link strength (TLS) was used for the quantitative evaluation of chains. A similar analysis was used to create a visual map of countries or regions, organizations, and authors published in each period to map the evolution of these elements (12). CiteSpace is an important bibliometric analysis software (13) that can realize the understanding of the main fields, research hotspots, and frontiers of fetal therapy.

## 3 Results

### 3.1 Annual global publication output and citations

**Figure 2:**
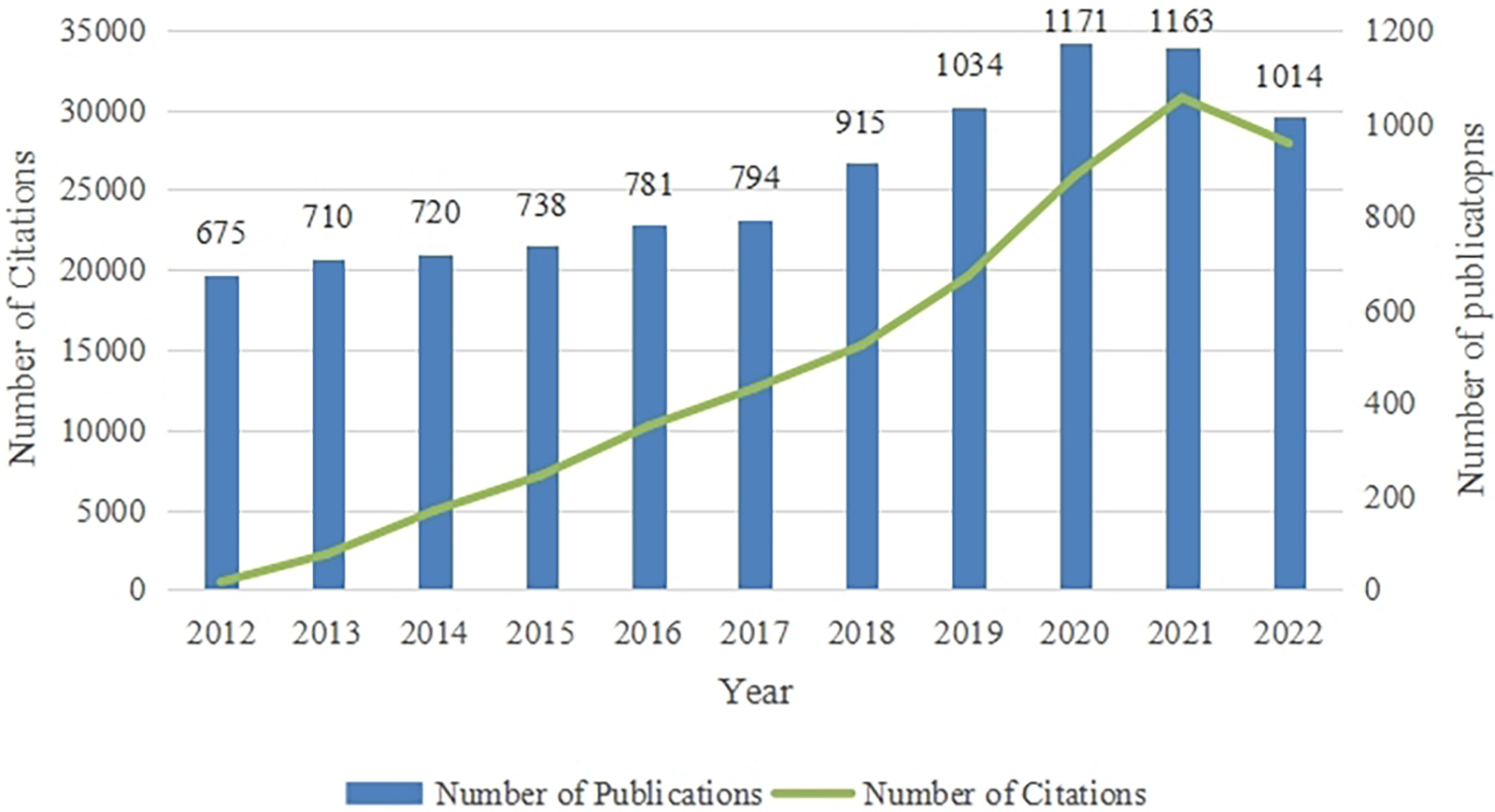
Trends in annual citations and publications about fetal therapy from 2012 to 2022.

A total of 9,715 articles related to fetal therapy from 2012 to 2022 were retrieved from the WOS Core Collection. The outputs of fetal therapy published annually are shown in Figure 2. Interest in intrauterine interventions has increased dramatically over the past 10 years. The annual number of publications worldwide increased from 675 in 2012 to 1,171 in 2020. The number of articles published in 2021 and 2022 were slightly lower than those in previous years; however, the number of articles was still increasing. The citation frequency was also an important indicator for academic quantitative evaluation. During the last decade, the citation frequency of the study annually increased and it was the highest in 2021. In the past decade, the number and citation frequency of articles in the field of fetal therapy have been increasing, which indicates that research in this field is in a booming period.

### 3.2 Analysis of countries

**Figure 3:**
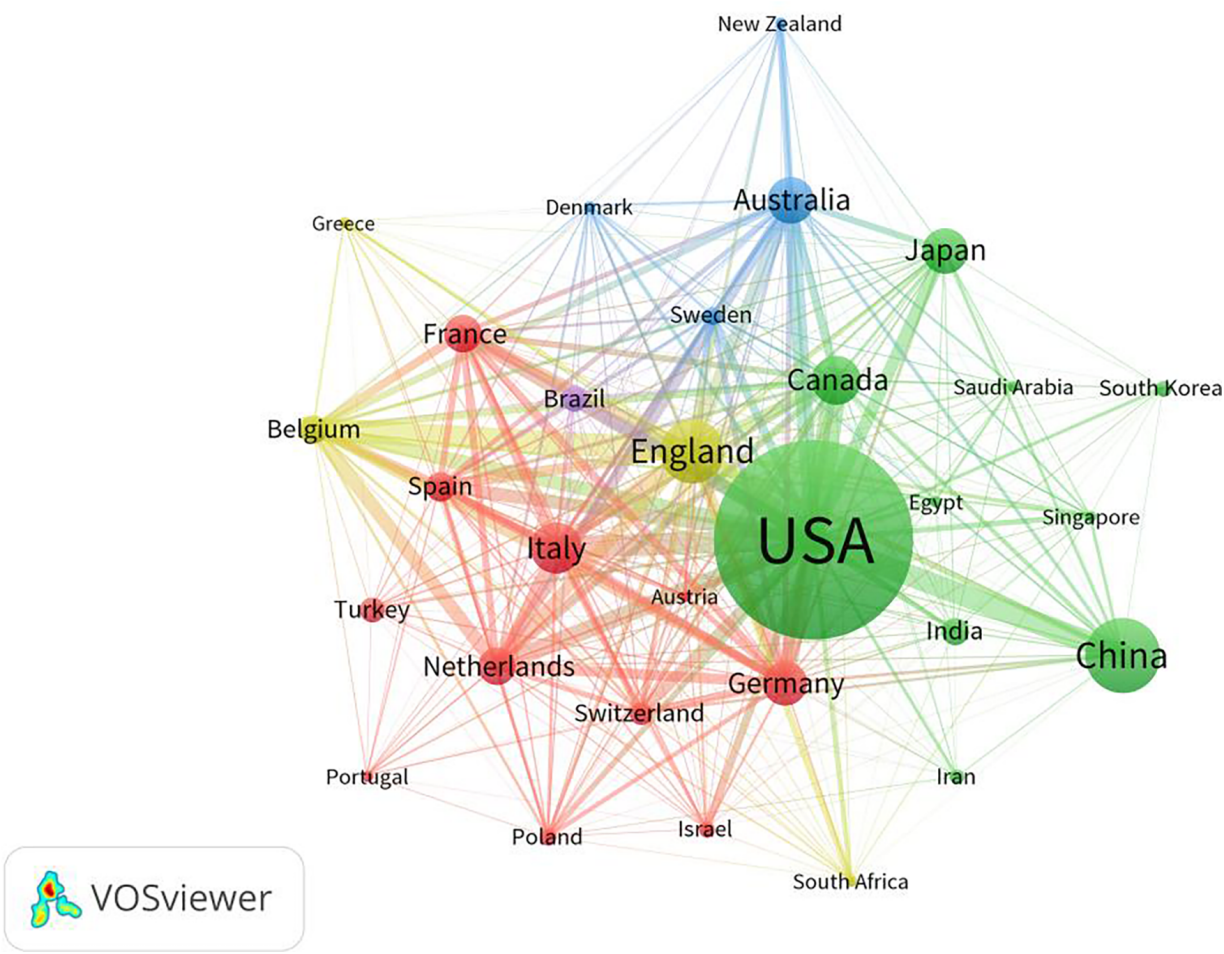
Map network of top 30 countries. Different colors indicate different clusters, and the size of circles indicate the number of publications. The thickness of the lines represents the link strength of the countries.

The number of publications in a country reflects the level and effect of research on the relevant field in that country. A total of 9,715 articles were published in the field of fetal therapy from 133 countries/regions. Table 1 lists the top ten countries with the highest number of papers. As shown in Table 1, the top three countries were the United States (3,334), China (987), and the United Kingdom (803), and these countries accounted for >50% of the publications and made important contributions to the research in this field. Regarding the number of citations, the top three countries were the United States, the United Kingdom, and Australia. The United States ranked first in the number of publications and citations in this field. This indicates its important role in fetal therapy and provides many excellent articles for researchers in the same field. Figure 3 illustrates the top 30 countries with >70 published articles generated via VOSviewer. As shown in this figure, research forces are mainly concentrated in the United States, China, United Kingdom, Italy, Canada, and Australia. Moreover, countries maintain a relatively close cooperative relationship, which also greatly promotes the continuous development of fetal therapy.

**Table 1:** Top 10 countries about fetal therapy.

Rank	Country/region	Documents	Citations	Total link strength
1	USA	3334	69,864	1,555
2	China	987	11,667	298
3	England	803	19,765	1,022
4	Italy	622	12,004	594
5	Canada	580	11,761	552
6	Australia	557	12,649	572
7	Japan	542	6,522	240
8	Germany	522	11,548	631
9	Netherlands	422	11,320	627
10	France	420	9,348	473

### 3.3 Analysis of institutions and authors

In total, 345 institutions have published research articles on fetal therapy. Table 2 lists the top ten institutions with the largest number of publications on fetal therapy. The top three institutions were University of Pennsylvania, the University of Toronto, and University College London. Four of the top ten institutions are located in the United States, two in Australia, and the rest in Canada, the United Kingdom, Netherlands, and Belgium, confirming the pre-eminent position of the United States, the United Kingdom, Australia, and Canada in this field. Interestingly, regarding the number of citations, the top four institutions were University of Pennsylvania,University College London, Leiden University, and the University of Toronto, which publish articles that receive more attention from researchers in the same field. In terms of total connection strength, Katholieke University of Leuven, University of Pennsylvania, and the University College London interacted more frequently with other research institutions.

**Table 2:** Top 10 institutions about fetal therapy.

Rank	Institution	Country	Documents	Citations	Total link strength
1	University of Toronto	Canada	214	3,870	158
2	University College London	United Kingdom	189	4,441	166
3	The Children's Hospital of Philadelphia	USA	176	3,565	201
4	Baylor College of Medicine	USA	170	2,850	161
5	University of San Francisco	USA	163	3,769	142
6	Leiden University	United Kingdom	161	4,309	75
7	Monash University	Australia	158	3,361	93
8	Katholieke Universiteit Leuven	Belgium	147	2,567	213
9	University of Pennsylvania	USA	136	3,086	167
10	The University of Melbourne	Australia	124	3,025	75

**Figure 4:**
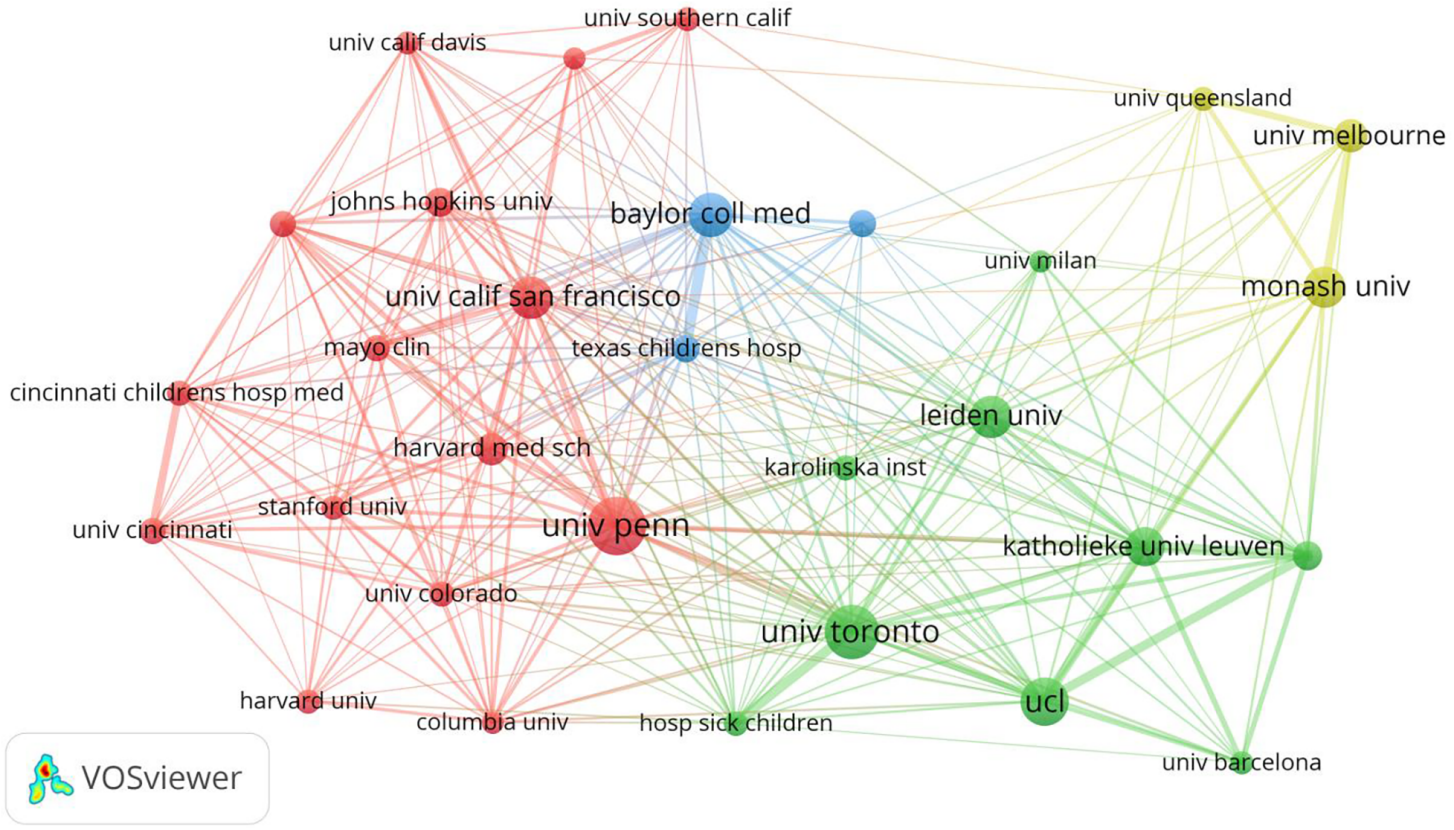
Map network of top 30 institutions. Different colors indicate different clusters, and the size of circles indicate the number of publications. The thickness of the lines represents the link strength of the institutions.

Figure 4 is a visual map generated via VOSviewer for institutions with >74 published articles. As shown, the main research power in this field is concentrated in University of Pennsylvania, University of Toronto, University College London, Baylor College of Medicine, and University of San Francisco.

Table 3 lists the most influential experts in fetal therapy in the last decade, ranked by the number of publications. The top three citations were Deprest J, Chmait RH and Oepkes D, Deprest J, Adzick NS and Oepkes D, in this order. Statistically, Deprest J ranked first in both the number of publications and the number of citations, indicating that his outstanding contributions to fetal therapy have been recognized by researchers in the same field.

**Table 3:** Top 10 authors about fetal therapy.

Rank	Author	Documents	Citations	Total link strength
1	Deprest J	102	1,677	48
2	Chmait RH	63	508	62
3	Oepkes D	62	1,115	50
4	Flake AW	54	895	54
5	Lopriore E	52	935	51
6	Ruano R	50	599	39
7	Adzick NS	48	1,622	54
8	Belfort MA	48	927	68
9	David AL	47	779	25
10	Gratacos E	43	769	16

### 3.4 Analysis of keywords

**Figure 5:**
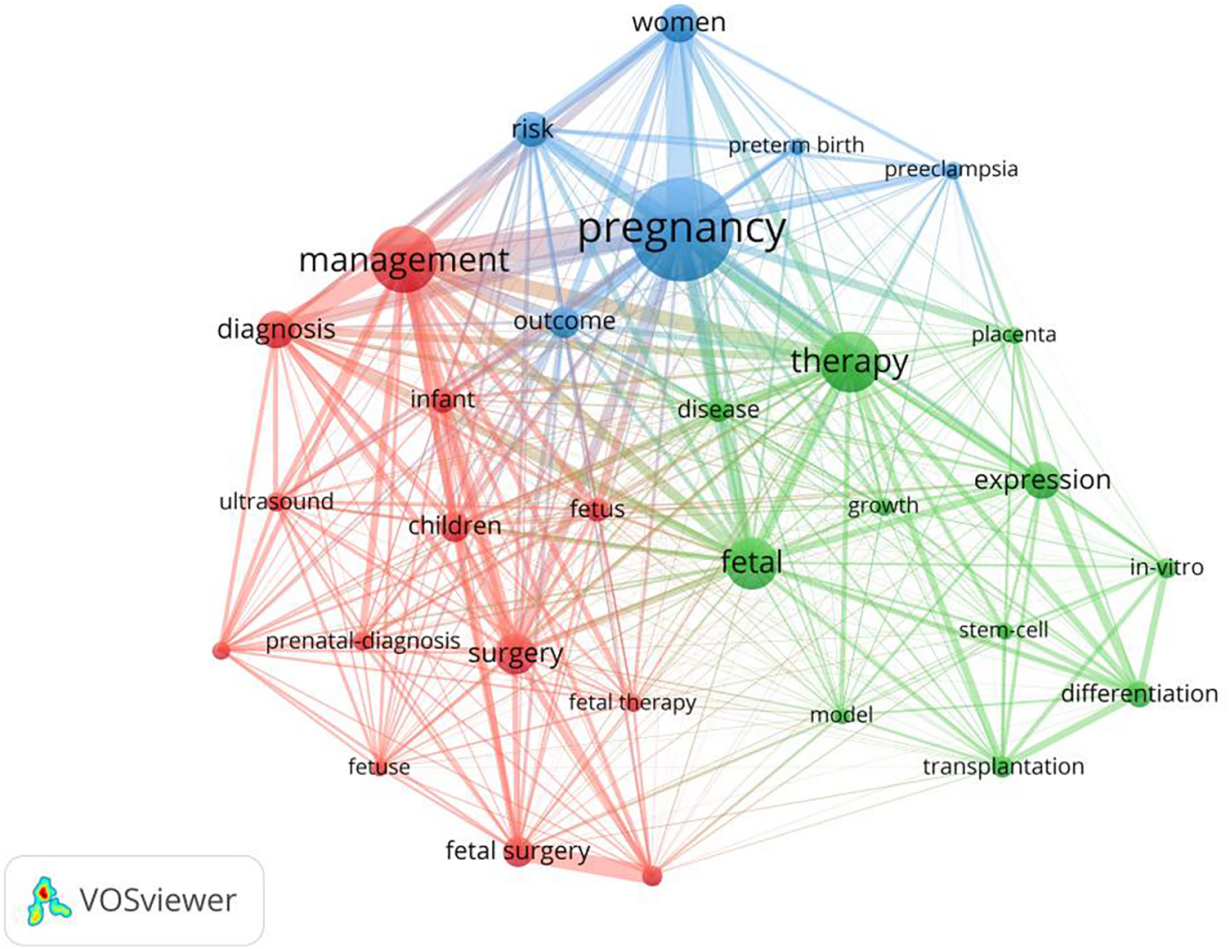
Map network of top 30 keywords. Different colors indicate different clusters, and the size of circles indicate the number of publications.

The analysis of keyword co-occurrence provides a theoretical basis for the in-depth understanding of the distribution and evolution of various research hotspots within a topic. As a highly simplified form of the content of the paper, keywords can directly and simply express the topic to a certain extent. Keywords selected by the author when submitting the manuscript for publication were extracted using VOSviewer. We analyzed keywords extracted from 9,715 publications. Figure 5 shows detailed data on the co-occurrence of the top 30 keywords. Among them, “management,” “prenatal diagnosis,” “fetal surgery,” and related keywords are the most significant.

**Figure 6:**
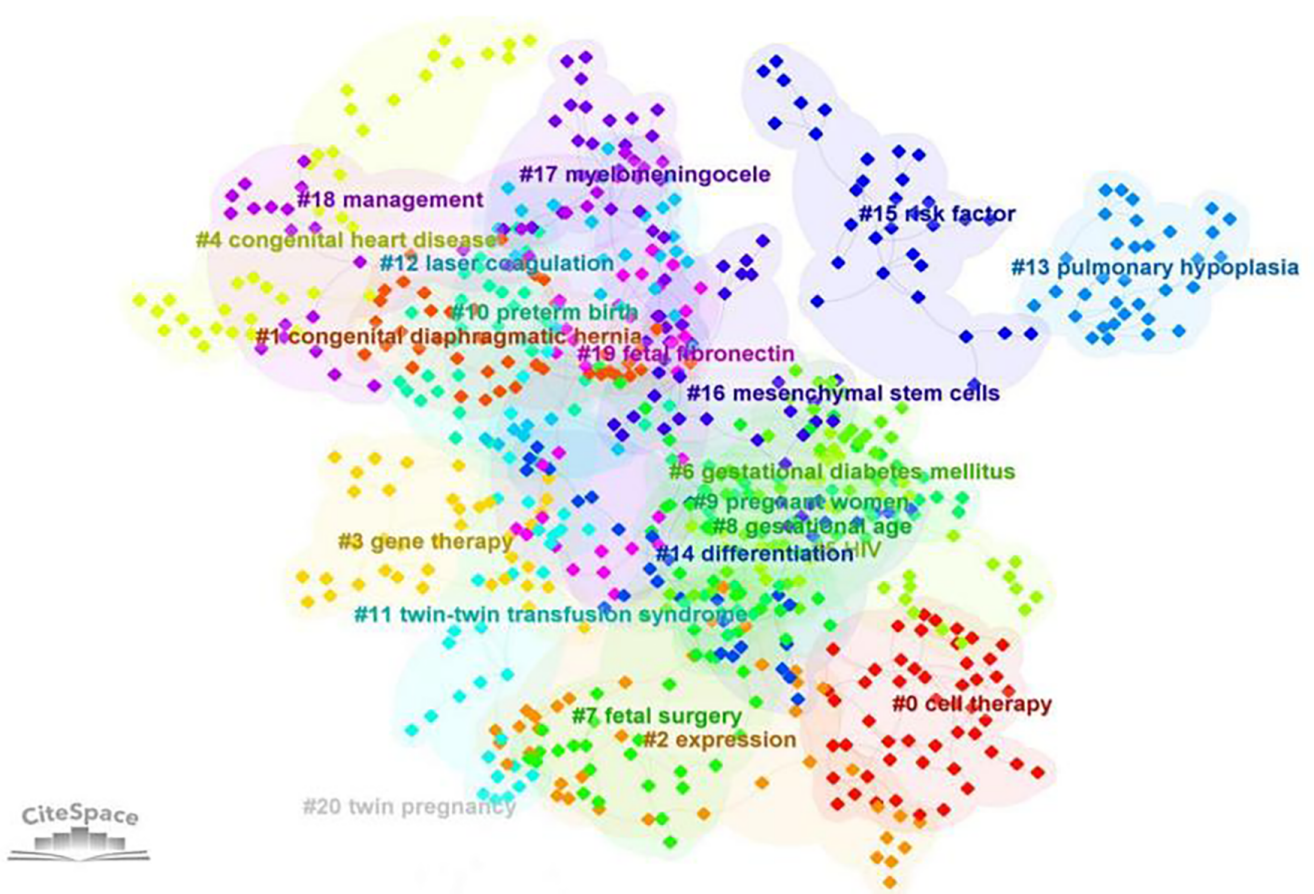
Clustering analysis of keywords (the order is from 0 to 22; the smaller the number, the more keywords the cluster contains, and therefore the more attention it receives).

As shown in Figure 6, in the keyword cluster analysis, keywords were divided into 20 clusters, including #0 cell therapy, #1 congenital diaphragmatic hernia, #2 expression, #3 gene therapy, #4 congenital heart disease, #5 HIV, #6 gestational diabetes mellitus, #7 fetal surgery, #8 gestational age, #9 pregnant women, #10 preterm birth, #11 twin–twin transfusion syndrome, #12 laser coagulation, #13 pulmonary hypoplasia, #14 differentiation, #15 risk factor, #16 mesenchymal stem cells, #17 myelomeningocele, #18 management, #19 fetal fibronectin, and #20 twin pregnancy.

**Figure 7:**
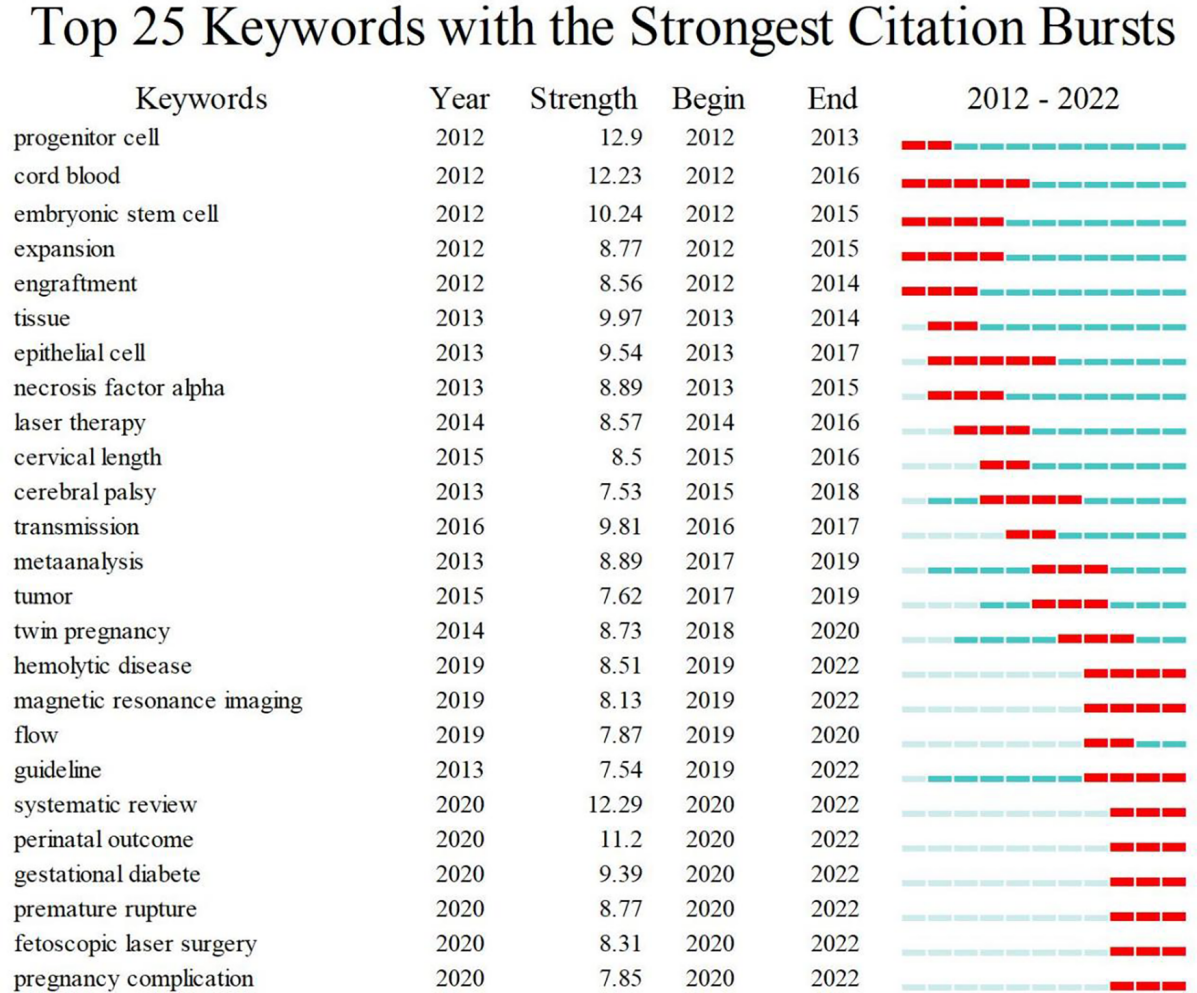
Top 25 keywords with the strongest citation burst in the fetal therapy field.

CiteSpace was used to detect frequently occurring words, known as citation burst keywords, which can reflect cutting-edge topics and research development. Figure 7 shows the top 25 keywords with the strongest citation burst. The keyword burst analysis continued until 2022, indicating the hotspots in fetal therapy research. Of the 25 keywords detected, magnetic resonance imaging (MRI), guideline, and fetoscopic laser surgery are hot topics in recent years.

### 3.5 Analysis of cocited references

**Figure 8:**
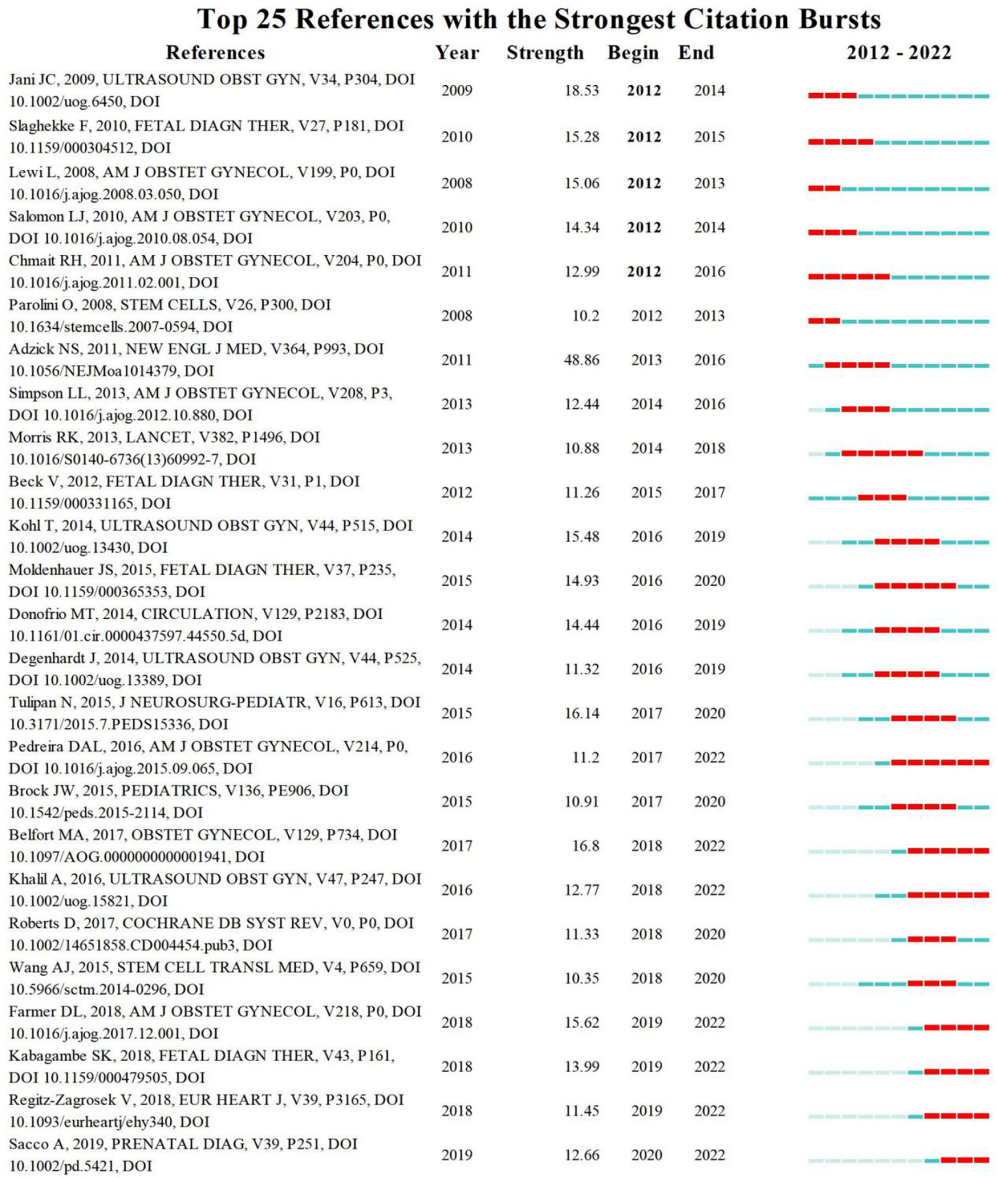
Top 25 references with the strongest citation burst in the fetal therapy field.

Cocitation analysis focused on research topics that were closely related to a particular field. To further explore its development trend, Table 4 lists our top ten cited studies during the study period. Overall, articles have low citation rates when newly published; thus, the current citation count may lag behind the actual value of articles. We also identified the top 25 articles with the strongest citation outbreak (Figure 8), which is considered an indicator of research frontiers or emerging trends. Combining these two study types helped identify key studies that influenced developments in fetal therapy.

**Table 4:** Top 10 most highly cited publications.

Rank	Title	Author	Year	Journal	Frequency
1	A randomized trial of prenatal versus postnatal repair of myelomeningocele	Adzick NS	2011	N Engl J Med	141
2	Fetoscopic open neural tube defect repair: development and refinement of a two-port, carbon dioxide insufflation technique	Belfort MA	2017	Obstet Gynecol	103
3	Endoscopic surgery for the antenatal treatment of myelomeningocele: the CECAM trial	Pedreira DAL	2016	Am J Obstet Gynecol	73
4	Fetal myelomeningocele repair: the post-MOMS experience at the Children's Hospital of Philadelphia	Moldenhauer JS	2015	Fetal Diagn Ther	68
5	The management of myelomeningocele study: full cohort 30-month pediatric outcomes	Farmer DL	2018	Am J Obstet Gynecol	68
6	Fetal surgery for myelomeningocele: a systematic review and meta-analysis of outcomes in fetoscopic versus open repair	Kabagambe SK	2018	Fetal Diagn Ther	61
7	The management of myelomeningocele study: obstetrical outcomes and risk factors for obstetrical complications following prenatal surgery	Johnson MP	2016	Am J Obstet Gynecol	59
8	Fetoscopic laser coagulation of the vascular equator versus selective coagulation for twin-to-twin transfusion syndrome: an open-label randomised controlled trial	Slaghekke F	2014	LANCET	57
9	Prenatal surgery for myelomeningocele and the need for cerebrospinal fluid shunt placement	Tulipan N	2015	J NEUROSURG-PEDIATR	56
10	ISUOG practice guidelines: role of ultrasound in twin pregnancy	Khalil A	2016	ULTRASOUND OBST GYN	54

## 4 Discussion

### 4.1 General information

We selected 9,715 articles on fetal therapy from January 2012 to December 2022 for bibliometric analysis and visualization and used study-retrieval programs VOSviewer and CiteSpace to obtain a more accurate and clear understanding of the latest research trends in this field. During the studied decade, the number of research articles on fetal therapy has steadily increased steadily, and the number of citations has also shown an obvious upward trend. Information on the characteristics of the study areas can be obtained from the country/region, institution, and author analysis. The main body of fetal therapy research is primarily concentrated in the United States, China, Britain, and other countries, and the fetal research centers in these countries are the main force of published studies in this field. In the author analysis, not much difference was observed in the number of published articles, which indicates a large space for exploration. Currently, most fetal studies are single-center studies with less cross-institutional communication. In the future, fetal studies should gradually move toward multicenter research.

The top ten cocited literature mainly included myelomeningocele surgeries, fetal laser treatment of twin transfusion syndrome, and ultrasound examination of twin pregnancy. The fetal surgeries related to myelomeningocele and twin transfusion syndrome are more mature in clinical application, twin ultrasound examination is completely different from that performed in single pregnancies, and prenatal monitoring is needed to avoid adverse complications.

By showing the co-cited references, the relationship between many topics in the discipline is sorted, and the reader is more familiar with it. From the strongest citation burst, the top 25 articles reflect the mature clinical application of diseases in fetal therapy, and the twin–twin transfusion syndrome, myelomeningocele, diaphragmatic hernia, and fetal congenital heart diseases are still the hotspots of research improvement in recent years. To avoid complications following treatment, cell therapy has gradually entered the field of view of researchers and become a hot research topic in recent years. By combining the most frequently cited references with the strongest citation burst, high values that contribute to the field of fetal therapy were identified. It is an essential reference for researchers who want to understand fetal therapy.

### 4.2 Keywords

Our keyword clusters cover the influence of maternal factors on fetal therapy, indications for fetal therapy, therapeutic means, and complications of fetal therapy, and these are major research topics in this field.

Fetal surgery, gene therapy, and stem cell therapy were the main fetal therapies mentioned in the keyword analysis in the past ten years. Fetal surgery, as a form of correction treatment, is a major topic in fetal therapy (14–26). At present, more mature fetal surgical methods include open hysterotomy, small-access hysteroscopy, and image-guided percutaneous fetal access (27), which have been applied to diseases such as twin–twin transfusion syndrome, thoracic malformations, fetal airway obstructions, myelomeningocele, and aortic valve stenosis (28). Although problems such as premature rupture of membranes and fetal distress may occur after invasive treatment (29), the benefits of fetal treatment against those of congenital diaphragmatic hernia, severe congenital heart disease (30–32), myelomeningocele, and twin transfusion syndrome must be evaluated to determine the fetal treatment that has achieved some success. The benefits of fetal surgical treatment outweigh the risks associated with treatment, resulting in increased survival and improved organ function. Surgical treatment of severe congenital diaphragmatic hernia can significantly improve the survival rate at discharge (33–37). The modified laser surgery for twin–twin transfusion syndrome has reduced complications associated with previous conventional laser treatment (38), and surgical repair of a fetus with myelomeningocele has led to a significant reduction in hindbrain herniation, decrease in shunt-dependent hydrocephalus, and improvement in lower-extremity motor function (39, 40).

Accordingly, gene therapy and cell therapy have become new hot topics (41–43). Gene therapy has yielded satisfactory results in theoretical studies on animal models of hemophilia, muscular dystrophy, central nervous system disease, cystic fibrosis, and other pulmonary diseases (44). Maternal risk from prenatal gene therapy is more dependent on the carrier used and the mode of administration.So far, animal studies have not demonstrated any significant germline or maternal effect of prenatal gene therapy (45). This has considerably helped gene therapy research advancement and exploration.

In cell therapy, hematopoietic stem cell research (46) is in the stage of animal models; however, it has successfully overcome important obstacles such as engraftment and immune barriers (47). It possesses the potential to be used in the clinical treatment of fetal anemia in the future. Prenatal mesenchymal stem cell (MSC) transplantation has been used to treat fetal osteogenesis imperfecta in humans, which is characterized by lower levels of bone implantation and improved linear growth, mobility, and fracture rates after treatment, compared with other patients with osteogenesis imperfecta. These favorable results make fetal MSC transplantation a promising option for clinical applications (48). Furthermore, in an animal model that is prone to preterm birth and premature rupture of membranes after surgery, transamniotic stem cell therapy enhanced postoperative recovery to a greater extent by promoting local tissue regeneration at the surgical site (49). The use of biological cells or collagen replacement drugs can also reduce the incidence of complications of inclusion body cysts and myelomeningocele surgery (50), which has implications for the perfection of myelomeningocele surgery.

The most common complications of fetal surgery are premature delivery, premature rupture of membranes,and placental abruption, etc. In recent years, the artificial uterus (AU) has also introduced brought a new perspective to fetal therapy. AU attempts to mimic the human nature environment of the human uterus and thus, provides an alternative for placental insufficiency or high-risk pregnancy caused by premature membrane rupture. For example, AU supports the lungs to mature to the extent where independent normal breathing is achieved, avoids immature development, and infection, and reduces the incidence of premature brain injury, such as intracranial hemorrhage in preterm infants. Interestingly, AU has social benefits as well. AU enables infertile couples and women who have undergone hysterectomy to achieve pregnancy. Thus, the AU is a useful surrogate alternative. However, AU cannot fully replicate the role of placenta in fetal development, such as gas exchange, metabolic waste product clearance, nutritional support, development of temporary immune system, production of various hormones, enzymes and cytokines to support the growth of the fetus. Thus, AU cannot completely duplicate uterine function, but it can be considered as a type of fetal treatment.

### 4.3 Research hotspots and frontiers

Keywords with strong burst strength are implicated as those that received special attention by the scientific community during a specific period and could therefore represent research hotspots and frontiers of a special field or subject in one period. Therefore, three research hotspots were selected for in-depth analysis, namely, fetoscopic laser surgery, guideline, and MRI, which are the current research frontiers.

At present, fetal laser therapy for twin–twin transfusion syndrome (51) is relatively mature; however, postoperative neurodysplasia may occur (52, 53), which still needs further improvement. Over the past decade, fetal laser therapy for chorionic angioma, sacrococcygeal teratoma, lower urinary tract obstruction, chest mass, and airway obstruction have emerged, and more post-treatment data are needed in the future to determine whether fetal laser therapy is a more beneficial treatment option (54–56).

Moreover, magnetic resonance examination, as an effective supplement to prenatal ultrasound diagnosis, can reflect the complex anatomical structure more directly and play an important role in late fetal imaging (57). It has contributed to the determination of fetal intrauterine intervention indications, intervention program selection, organ function evaluation, and so on. For example, in severe congenital heart disease, MRI can accurately reflect the fetal heart anatomy, tetralogy of Fallot in the absence of a pulmonary valve, severe Ebstein malformations with heart failure, or in some cases severe pulmonary comorbidities due to bronchial or lung compression or lung dysplasia. It can be used for fetal risk assessment and management planning (30), and additional information on fetal circulation distribution and fetal oxygen transport can be obtained, which opens the possibility for minimally invasive fetal cardiac interventional therapy and the development of fetal management strategies with neuroprotective effect (58). The research focuses on T1- and T2-weighted imaging, diffusion tensor imaging, quantitative imaging of T1/T2 relaxation time, myelin water fraction, and magnetization transfer ratio in nervous system diagnosis. Other emerging MRI techniques, such as magnetic sensitive mapping and phase imaging, are expected to help characterize the microstructure of the developing white matter (59, 60). In the digestive system, MRI allows for the visualization of the extent and pattern of dilated bowel loops (61). In the skeletal musculoskeletal system, the length and morphology of the ossified bone can be assessed, and three-dimensional imaging can provide a comprehensive assessment of fetal bone morphology. Dynamic MRI helps describe distal limb abnormalities, fetal dyskinesia, and contraction (62).

The American College of Obstetricians and Gynecologists, American Academy of Pediatrics, and Maternal-Fetal Medical Society regularly issue guidelines on the screening, diagnosis, and treatment of fetal diseases to provide standardized methods for the prenatal diagnosis and management of fetal diseases, achieve standardized and safe diagnosis and treatment, and maximize clinical care. Continuous updating of clinical guidelines in fetal therapy also prompts researchers to pay attention to recent problems and make targeted exploration to achieve qualitative breakthroughs in fetal therapy.

HIV is commonly used as an exclusion criterion in studies on fetal treatment. Elrod J (63) attempted to repair fetal spina bifida in a pregnant woman with HIV infection, and the fetus was confirmed to be free of HIV infection after birth. This case report also brought some inspiration to fetal treatment. While emphasizing the progress of overall medical research, we should also monitor progress in individualized medicine, rather than strictly following outdated standards.

The effective control of diseases during pregnancy is also an important aspect of protecting the fetus. The specificity of prenatal treatment lies in the relationship between the mother and fetus. When treating the fetus, the safety of the pregnant woman should also be ensured, and the negative effect on the fetus should also be ensured in the control of pregnancy diseases. For example, zidovudine as an antiretroviral therapy during pregnancy affects fetal cardiac function (64, 65), glyburide has limited control of gestational diabetes mellitus, and metformin is associated with preterm birth (66–71). Angiotensin-converting enzyme inhibitors should be avoided for hypertension in early pregnancy (72, 73).

The research content and hotspots in the past decade reflect the gradual progress of fetal therapy; thus, more fetal therapy methods will be applied to clinical practice in the future.Based on the frequency of the keywords (Table 5), we listed the current fetal diseases in fetal therapy to provide clarity on the diseases that concern fetal therapy research.

**Table 5:** The frequency of fetal disease keywords.

Frequency	Keyword	Year	Frequency	Keyword	Year
405	Twin-twin transfusion syndrome	2012	43	Urinary tract obstruction	2014
273	Congenital diaphragmatic hernia	2012	32	Chiari ii malformation	2017
231	Spina bifida	2012	27	Twin anemia polycythemia sequence	2013
227	Congenital heart disease	2012	27	Posterior urethral valve	2014
184	Neural tube defect	2012	21	Cardiac arrhythmia	2014
126	Bronchopulmonary dysplasia	2012	19	Congenital heart block	2013
103	Myelomeningocele	2014	16	Sacrococcygeal teratoma	2013
79	Pulmonary hypertension	2012	5	Airway obstruction syndrome	2013
68	Cerebral palsy	2012	4	Bronchopulmonary sequestration	2017
66	Fetal anemia	2012	4	Fetal alcohol syndrome	2012
59	Cystic adenomatoid malformation	2012	4	Abdominal wall defect	2018

## 5 Limitations

This study still has some limitations. First, data sources were limited to the WOS. Given the limitations of existing software, directly combining search results from multiple databases is not possible. We used WOS instead of other databases, such as Embase and PubMed. It is recognized as the most commonly used and suitable bibliometric database in scientometrics in a format that can be directly recognized by the metrological software used in this study. In addition, the language was limited to English and therefore did not include few multilingual studies. The effect of recently published high-quality articles may be underestimated because they may not have had enough time to accumulate enough citations. A follow-up study is necessary to assess the effect of these articles on the field. This study analyzed the research on fetal therapy in the past decade and summarized the current status and research trends in this field, which is believed to provide some reference for future studies.

## 6 Conclusion

To our knowledge, this study provides the first multidimensional analysis of fetal therapy studies from a bibliometric perspective. By reviewing the publications in the past decade, this bibliometrics visually presents fetal therapy research, including publication trends, institutions, authors, and country analyses, summarizes research content, and identifies research hotspots. These results allow the research community to identify emerging themes and frontiers to guide future fetal therapy research.

## Data Availability

The raw data supporting the conclusions of this article will be made available by the authors, without undue reservation.
